# Human Case of *Atopobium rimae* Bacteremia

**DOI:** 10.3201/eid1502.071399

**Published:** 2009-02

**Authors:** Emmanouil Angelakis, Véronique Roux, Didier Raoult, Michel Drancourt

**Affiliations:** Université de la Méditerranée, Marseille, France

**Keywords:** Atopobium rimae, bacteremia, E-test, fever, letter

**To the Editor:** The genus *Atopobium* (*1*) accommodates species formerly designated *Lactobacillus minutus*, *L. rimae,* and *Streptococcus parvulus* (*2*). Use of 16S rDNA sequence analysis showed these species to be closely related and to form a distinct line of descent within the lactic acid bacteria (*3*). *Atopobium* spp. usually have been recognized as part of the human gingival oral flora; some species, including *A. rimae* and *A. parvulum*, have been identified as agents of chronic periodontitis (*4*,*5*). *A. rimae*, formerly known as *L. rimae* (*1*), forms short, gram-positive, strictly anaerobic, elliptical bacteria with low G+C content (*4*). *A. rimae* produces large amounts of lactic acid and has been recovered previously from normal human gingival flora (*4*,*5*). Apart from periodontitis, it has not been implicated in other types of infection. We report an unusual case of *A. rimae* bacteremia.

In May 2007, a 77-year-old woman with a history of right thoracotomy for pneumothorax 2 years earlier was hospitalized for inhalation pneumonia caused by paralysis of the right vocal cord. During hospitalization, septic shock and a fever of 38°C developed in the patient, complicated by acute respiratory failure and stroke. She was transferred to an intensive care unit with a PaO_2_/FiO_2_ >300 mm Hg, and a tracheotomy was performed. Three anaerobic blood specimens, drawn at entrance into the intensive care unit, yielded gram-positive cocci after 24-h incubation of the first bottle and gram-positive bacilli after 48-h incubation of the 2 other bottles. The gram-positive cocci were identified as *Streptococcus gordonii* using API STREP (bioMérieux, Marcy l’Etoile, France). The gram-positive bacilli were catalase negative and oxidase positive but remained unidentified with use of API ANA strip (bioMérieux). Minimum inhibitory concentrations of antibiotics were determined for the gram-positive bacilli using E-test assay (AB BIODISK, Solna, Sweden) on Columbia agar supplemented with 5% sheep blood. Minimum inhibitory concentrations were 0.064 μg/mL for penicillin G, 0.023 μg/mL for ampicillin, 0.012 μg/mL for amoxicillin–clavulanic acid, 0.032 μg/mL for imipenem, <0.016 μg/mL for azithromycin, <0.016 μg/mL for erythromycin, 0.06 μg/mL for ciprofloxacin, and 1.25 μg/mL for vancomycin. DNA was extracted from 1 colony by using a QIAamp tissue kit (QIAGEN, Hilden, Germany) as described by the manufacturer. The 1,454-bp 16S rDNA sequence obtained using the fD1 5′-AGAGTTTGATCCTGGCTCAG-3′ and rP2 5′-ACGGCTACCTTGTTACGACTT-3′ primer pair (*6*,*7*) showed 99% sequence similarity with the 16S rDNA sequence of *A. rimae* (GenBank accession no. AF292371) by use of BLAST version 2.2.9 software (National Center for Biotechnology Information). A phylogenetic neighbor-joining tree based on the *Atopobium* spp. 16S rDNA sequences made with the MEGA software confirmed that the isolate belonged to *A. rimae* (Figure). Initial treatment by intravenous tazocilline-amikacin was changed to intravenous amoxicillin–clavulanic acid (2 g/200 mg). The fever resolved, and the patient’s condition improved. The treatment was stopped after 7 days, and the patient remained apyretic.

**Figure:**
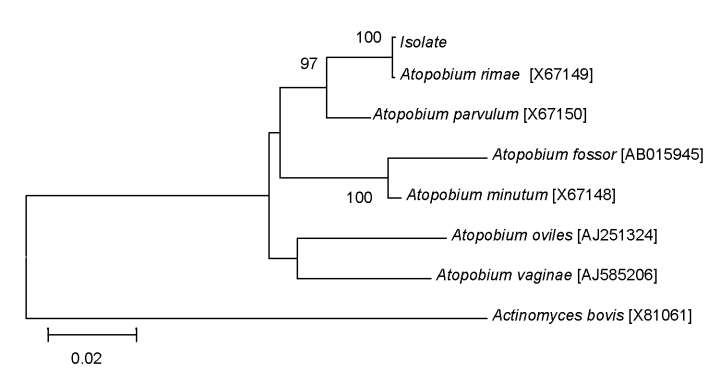
16S rDNA maximum-likelihood phylogenetic tree showing the relationships of a blood isolate with *Atopobium* species. GenBank accession numbers are indicated in brackets. 16S rDNA sequence of *Actinomyces bovis* was used as an outgroup. Bootstrap values >90% as indicated at nodes. Scale bar indicates 0.02 substitutions per nucleotide position.

In this case, phenotypic identification of gram-positive bacillus isolated from 2 blood cultures failed because the definite bacterial species *A. rimae* was not included in the API database used for the phenotypic identification. Final identification was achieved within 2 days by comparison of the almost complete 16S rDNA sequence with homologous sequences deposited in Genbank. This comparison yielded a 99% sequence similarity, regarded as criteria for accurate identification of bacterial organisms at the species level (*8*). In this patient, 2 *A. rimae* isolates were recovered from 2 different blood-culture bottles drawn 48 h apart, suggesting that *A. rimae* was not just a bypassing organism but indeed responsible for septicemia. In these specimens, *S. gordonii* was also isolated. Both species have been described as belonging to the oral flora, suggesting that these flora probably were the source for mixed septicemia in the patient. *A. rimae* was isolated as the patient was presenting with clinical features of septic shock, suggesting that *A. rimae* may have contributed to the shock. Antimicrobial drug treatment based on in vitro *A. rimae* susceptibility profile, along with reanimation measures, allowed for the patient’s recovery.

This case report illustrates the usefulness of 16S rDNA sequencing for accurate identification of anaerobic organisms and suggests that *A. rimae* should be added to the list of organisms responsible for bacteremia in patients.
